# Investigation of measles outbreak-Herena and Dawe-Serer Districts of Bale Zone, Oromia Region, Ethiopia, February 2011

**DOI:** 10.1186/1742-4690-9-S1-P39

**Published:** 2012-05-25

**Authors:** Abyot Bekele Woyessa, Tesfaye Deti, Amanuel Yadata, Ashenafi Kenna, K Addisalem, M Yesuf, M Abebe

**Affiliations:** 1 Ethiopian Health and Nutrition Research Institute, Ethiopia, Addis Ababa, Ethiopia

## Introduction

An estimated 10 million cases and 164,000 deaths from measles occur worldwide each year. On 08 Feb 2011 Bale Zonal health department reported suspected measles outbreak. We investigated to identify the etiology of the outbreak and undertake appropriate prevention and control interventions.

## Materials and methods

Patient observation was made and active-cases were searched house to house. Medical registration-books were assessed and suspected measles cases were identified from 24-Nov-2010 to 15-Feb-2011 in Herena and Dawe-Serar districts using the following case definitions: maculopapular rash with fever ≥38.5°C with coryza, conjunctivitis or cough or epidemiologically linked by contact with laboratory confirmed outbreaks in neighboring districts. Immunization coverage and vaccine-storage facilities were assessed. Descriptive analysis was conducted using Epi-Info version3.5.1.

## Results

A total of 329 suspected measles cases and 30 community deaths (case fatality rate (CFR) 9.1%) were reported of which 159 (48.3%) were from Dawe-Serar and 170 (51.7%) were from Herena. CFR was 25/159 (15.7 %) in Dawe-Serar and 5/170 (2.9%) in Herena and higher among females than males (12.2% vs. 6.1%). All deaths and 140/329 (42.6 %) of the cases were not vaccinated against measles. Vaccination coverage was 45.4% in Dawe-Serer and 54% in Herena. The attack rate was highest among those 15 years of age. About 5/7 (71%) refrigerators used for vaccine-storage were not functional. Prior to investigation period, 110/159 specimens from 14 districts of the zone were tested positive for measles-IgM.

## Conclusions

An outbreak of suspected measles occurred in 2 districts affecting primarily those <5 years of age. Low-vaccination coverage and non-functional cold storage likely contributed to the outbreak. Undertaking supplementary vaccination activities, enhancing routine vaccination coverage and improvement in cold chain operation and maintenance need to be emphasized in the districts to reduce measles incidence.

